# Finding covert fluid: methods for detecting volume overload in children on dialysis

**DOI:** 10.1007/s00467-016-3431-4

**Published:** 2016-06-10

**Authors:** Marco Allinovi, Moin A Saleem, Owen Burgess, Catherine Armstrong, Wesley Hayes

**Affiliations:** 1 Bristol Children’s Renal Unit, Bristol Royal Hospital for Children, Upper Maudlin Street, Bristol, BS2 8BJ UK; 2 Paediatric Nephrology Unit, Meyer Children’s Hospital, Viale Pieraccini 24, 50141 Florence, Italy; 3 University of Bristol, Bristol, BS8 1TH UK; 4 Department of Paediatric Cardiology, Bristol Royal Hospital for Children, Upper Maudlin Street, Bristol, BS2 8BJ UK

**Keywords:** Fluid overload, Dialysis, Paediatrics, Bioimpedance, Echocardiography, Ultrasonography

## Abstract

**Background:**

Lung ultrasound is a novel technique for detecting generalized fluid overload in children and adults with end-stage renal disease (ESRD). Echocardiography and bioimpedance spectroscopy are established methods, albeit variably adopted in clinical practice. We compared the practicality and accuracy of lung ultrasound with current objective techniques for detecting fluid overload in children with ESRD.

**Methods:**

A prospective observational study was performed to compare lung ultrasound B-lines, echocardiographic measurement of inferior vena cava parameters and bioimpedance spectroscopy in the assessment of fluid overload in children with ESRD on dialysis. The utility of each technique in predicting fluid overload, based on short-term weight gain, was assessed. Multiple linear regression models to predict fluid overload by weight were explored.

**Results:**

A total of 22 fluid assessments were performed in 13 children (8 on peritoneal dialysis, 5 on haemodialysis) with a median age of 4.0 (range 0.8–14.0) years. A significant linear correlation was observed between the number of B-lines detected by lung ultrasound and fluid overload by weight (*r* = 0.57, *p* = 0.005). A non-significant positive linear correlation was observed between fluid overload by weight and bioimpedance spectroscopy (*r* = 0.43, *p* = 0.2), systolic blood pressure (*r* = 0.19, *p* = 0.4) and physical examination measurements (*r* = 0.19, *p* = 0.4), while a non-significant negative linear relationship was found between the inferior vena cava collapsibility index and fluid overload by weight (*r* = −0.24, *p* = 0.3). In multiple linear regression models, a combination of three fluid parameters, namely lung ultrasound B-lines, clinical examination and systolic blood pressure, best predicted fluid overload (*R* ^*2*^ = 0.46, *p* = 0.05).

**Conclusions:**

Lung ultrasound may be superior to echocardiographic methods and bioimpedance spectroscopy in detecting volume overload in children with ESRD. Given the practicality and sensitivity of this new technique, it can be adopted alongside clinical examination and blood pressure in the routine assessment of fluid status in children with ESRD.

## Introduction

Chronic fluid overload contributes to cardiovascular morbidity in children with end-stage renal disease (ESRD) [1–3]. Minimizing volume overload by optimizing the target weight in children’s dialysis prescriptions is therefore paramount. Prescription of target weight traditionally relies on clinical judgment together with the evaluation of parameters such as a history of intra-dialytic symptoms, inter-dialytic weight gain, physical examination and blood pressure. Practical tools to objectively assess fluid overload in infants and children with ESRD are needed to minimize chronic fluid overload and its associated morbidity.

A number of techniques are available to assess fluid overload in adults and children with ESRD, including bioimpedance spectroscopy (BIS) [4–7], echocardiographic assessment of inferior vena cava (IVC) dimensions [8, 9] and lung ultrasound [10–12]. The aim of this study was to evaluate the accuracy of these three techniques in detecting fluid overload in children with ESRD and to compare them with clinical measures, including weight, physical examination and systolic blood pressure.

## Methods

In this prospective observational study, all infants and children (age range 0–18 years) with ESRD receiving dialysis [haemodialysis (HD) or peritoneal dialysis (PD)] in our regional paediatric nephrology center between 1 May 2015 and 1 October 2015 were eligible to participate. Exclusion criteria were co-existent lung fibrosis, atelectasis, lymphangitis, interstitial lung disease, cardiac failure, acute respiratory distress syndrome or congenital cardiac anomalies. In patients with these conditions, pulmonary or cardiac pathology may confound the assessment of generalized fluid overload with lung ultrasound or echocardiography.

Thirteen children with a median age of 4.0 (range 0.8–14.0) years ultimately participated in the study, of whom five children received chronic HD and eight received PD for ESRD (Table 1). Four of the five children receiving HD were on the standard HD regimen of three sessions weekly, with each session 3–4 h long; the exception was one child who was receiving HD 4 times weekly for control of labile fluid status. The eight children on PD received automated PD 7 nights per week. Residual urine output was >25 ml/kg/day in seven patients, four patients were oliguric and two patients were anuric (Table 1).

**Table 1:** Patient characteristics at baseline

Patient characteristics	All patients (*n* = 13)	Peritoneal dialysis (*n* = 8 patients)	Haemodialysis (*n* = 5 patients)
Age (years)	4.0 (0.8–14.0)	2.0 (0.8–8.5)	6.0 (4.1–14.0)
Male gender	10 (77)	7 (87)	3 (60)
Duration of renal replacement therapy (months)	8.9 (1.3–41.4)	11.8 (1.3–41.4)	7.9 (4.0–11.5)
Residual urine output (good/oliguric/anuric)	7/4/2	3/3/2	4/1/0
Physical signs or symptoms of overt fluid overload	0	1 (12.5)	0
Hypertension requiring antihypertensive drugs:	5 (38)	3 (37.5)	2 (40)
Beta blocker	3 (23)	2 (25)	1 (20)
Alpha blocker	1 (8)	1 (12.5)	0
Calcium channel antagonist	4 (31)	4 (50)	0
Centrally acting	1 (8)	1 (12.5)	0

Data are presented as the median with the range in parenthesis or as the number of patients with the percentage in parenthesis, as appropriate

Baseline data were recorded at the time of entry to the study

Children receiving HD were assessed during routine in-center HD sessions, and those receiving PD were assessed at outpatient clinic reviews. In children with stable fluid status, a single fluid assessment was undertaken which included physical examination, weight, blood pressure, BIS, lung ultrasound and echocardiography measurements. Patients showing a significant variation in fluid status had two assessments performed at separate time points to reflect different degrees of fluid overload.

Clinical data included patient weight, blood pressure and physical examination findings. Patients were weighed undressed on a calibrated electronic scale. Blood pressure was measured consistent with international guidance [13]. The clinical impression of each patient’s fluid status was scored by the attending physician with −1 representing dehydration, 0 euvolaemia and 1, 2 and 3 representing mild, moderate and severe fluid overload, respectively. Clinical criteria for underhydration included systemic hypotension (blood pressure <5th percentile), symptoms of muscle cramps or dizziness and signs of cool peripheries, tachycardia, sunken eyes, reduced skin turgor or dry mucous membranes. Clinical criteria for fluid overload included interdialytic weight gain, oedema, increased jugular venous pressure, crackles on chest auscultation, low oxygen saturations, presence of a third or fourth heart sound and hypertension (blood pressure >95th percentile).

**Fig. 1:**
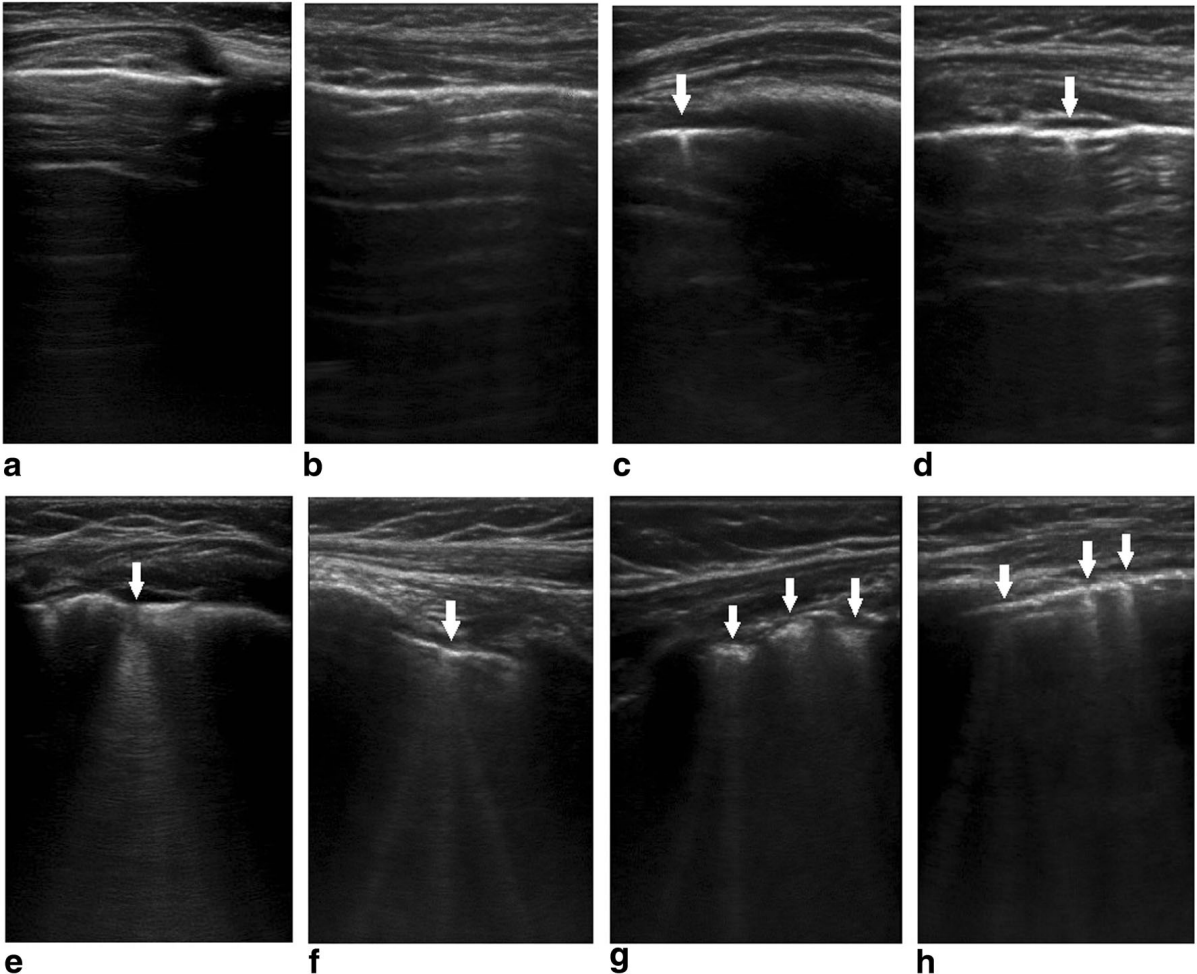
Lung ultrasound B-lines. **a**,** b** A-lines (parallel to the pleural line), **c**,** d** Z-lines (comet tails that do not obliterate A-lines and do not penetrate to the bottom of the ultrasound window;* arrows*), **e**,** f** single B-line (arising from a single point at the pleural line;* arrows*), **g**,** h** three B-lines (arising from three distinct points at the pleural line;* arrows*)

Lung ultrasound was used to quantify B-lines in infants and children using methodology previously reported [12]. All ultrasound examinations were performed at the bedside with a commercially available portable device (SonoSite S-ICU C60; SonoSite; Bothell, WA) equipped with a 6- to 13-MHz linear probe (L25x; SonoSite) in B-mode. B-lines are reverberation artifacts that originate at the pleural line, extend to the lower edge of the screen, move synchronously with pleural sliding and obliterate A-lines (Fig. 1). The total number of B-lines visualized in 28 intercostal positions was recorded. For children on HD, pre-dialysis examinations were performed within 15 min of starting the dialysis session, and post-dialysis assessments were undertaken within 15 min of completing the session. For children on PD, assessments were undertaken following nocturnal automated PD with an empty abdomen. Two operators performed the lung ultrasound assessments; both were blinded to BIS and echocardiographic measurements. Intra-observer variability between operators in ten independent lung ultrasound assessments was acceptable on Bland–Altman analysis [12].

Echocardiography was performed by one cardiologist using Vivid E9 equipment (General Electric Medical Systems, Milwaukee, WI). The operator was blinded to patients’ BIS and lung ultrasound measurements. Studies were performed in guided B- and M-mode with concurrent electrocardiography, with transducer frequencies appropriate for body size as per the recommendations of the American and European Society of Echocardiography for paediatric patients [14, 15]. Examinations were performed 15 min before dialysis sessions with the patients in a supine position. Echocardiograms were not performed after HD sessions due to the need to wait 2–3 h for vascular refilling; waiting after dialysis sessions was not acceptable to children and their parents. Echocardiographic measures included were IVC diameter during inspiration and expiration and collapsibility index. The maximum IVC diameter during expiration and the minimum diameter during deep inspiration were measured in long axis from the subxiphoidal position, 1.5 cm from the right atrial junction. Measurements were taken before the electrocardiographic P wave. The mean IVC diameter was calculated from the maximum IVC diameter during expiration and the minimum diameter during deep inspiration.

BIS was performed in children aged >2 years using a portable whole body BIS device (Body Composition Monitor; Fresenius Medical Care, Bad Homburg, Germany). This device uses 50 alternating current frequencies between 5 and 1000 kHz to measure body composition in relation to total body water, intracellular volume and extracellular volume as reflected by resistance to electric current. BIS was not performed on infants under 2 years of age as the technique has not been validated with appropriate reference algorithms in this age group. One physician took measurements prior to dialysis sessions. Patients were recumbent for at least 5 min prior to measurements, with arms and legs spread at an angle of approximately 30°. Children on PD lay supine with the abdomen drained. Four non-recyclable electrodes were attached to the hand and foot on the non-fistula side. If more than one assessment was undertaken, care was taken to fix electrodes in the same position for each measurement. Measurements with less than 85 % quality were discarded, and the mean of two sequential readings was used for analysis. Excess extracellular water was recorded both in litres and as a percentage of predicted values. Similar to echocardiography, post-dialysis BIS readings were not taken due to the need to wait 2–3 h for vascular refilling [16]; waiting after dialysis sessions was not acceptable to children and their parents.

Our ‘gold standard’ comparator was fluid overload by weight, expressed as the proportional (%) increase from the target weight. The target weight was that prescribed by the responsible physician using clinical data synthesized with trends in nutrition and growth. Systolic blood pressure elevation was expressed as the proportional (%) increase from the 50th blood pressure centile appropriate for age and height [13]. Echocardiographic measurements were normalized to patient body surface area [17] and compared to published reference ranges [18, 19]. Fluid overload derived from BIS was expressed as the proportional (%) overload relative to total body extracellular water.

The primary outcome was the correlation of technique-specific measurements with the increase in patient weight from baseline. Pairwise correlation between individual fluid overload parameters was determined using Pearson’s correlation. Agreement between fluid measurement techniques and the gold standard comparator, namely weight overload, was assessed using Bland–Altman analysis following natural logarithmic transformation of variables. Multiple linear regression models to predict fluid overload by weight were explored. Optimal variable selection for the regression model was undertaken using the leaps and bounds algorithm [20]. The frequency of dissociation between fluid overload measurements and systolic blood pressure was determined by the proportion of measurements suggesting overhydration in the face of blood pressure less than the 50th centile, or underhydration in the face of blood pressure greater than the 50th centile. All analyses were performed using STATA release 13 (StataCorp LP, College Station, TX)

## Results

A total of 22 fluid assessments were performed in 13 children. Two separate fluid assessments were performed in nine of these children, with each assessment representing different degrees of fluid overload. Single fluid assessments were undertaken in the remaining four patients, all of whom had stable fluid status and residual urine output of >25 ml/kg/day. At each assessment, all fluid-related variables were measured, with the exception of BIS, which was not performed in the two children aged <2 years. One child was obese with a body mass index of >95th centile for age. There were no associated technical difficulties in obtaining adequate lung ultrasound images to assess B-lines.

The children were assessed as clinically euvolaemic in 82 % of the physical examinations, as mildly clinically dehydrated in 14 % and as mildly overloaded in 4 %. No children were assessed as having moderate or severe clinical fluid overload at the time of the physical examination. Median fluid overload by weight for all assessments was 0.5 % (range −5.0 to 6.0 %). The median systolic blood pressure elevation above the 50th blood pressure centile was 6.4 % (range −20 to 52 %).

The median number of B-lines on the lung ultrasound image was four (range 0–26). The presence of up to five B-lines is considered to be a normal variant [21, 22]. Median indexed IVC diameters were 0.7 (range 0.2–1.0) cm/(kg/m^2^) during inspiration and 1.39 (range 0.6–2.1) cm/(kg/m^2^) during expiration. The median IVC collapsibility index was 50 % (range 25–83 %). BIS measurements showed a median overhydration of −0.6 % (range −4 to 2.0 %).

**Fig. 2:**
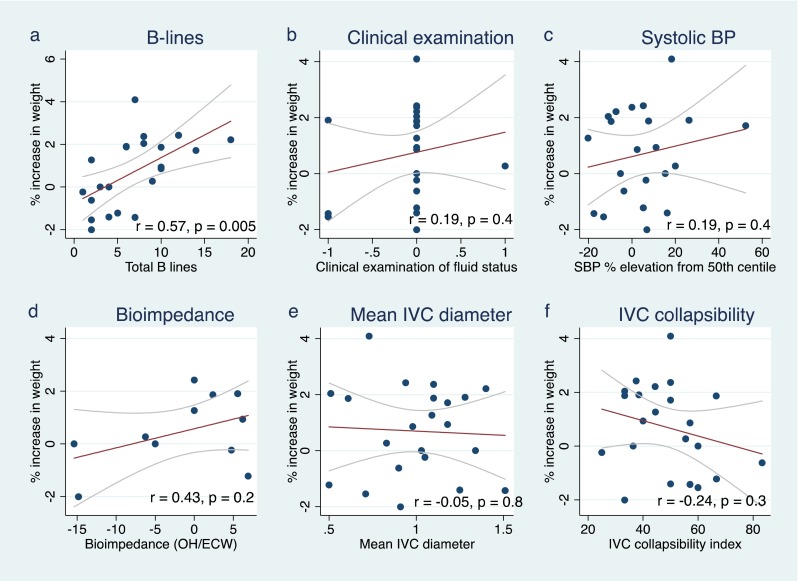
Correlation of fluid parameters with proportional increase in weight from the prescribed target weight.* SBP* Systolic blood pressure,* IVC* inferior vena cava,* OH/ECW* overhydration/extracellular water

**Fig. 3:**
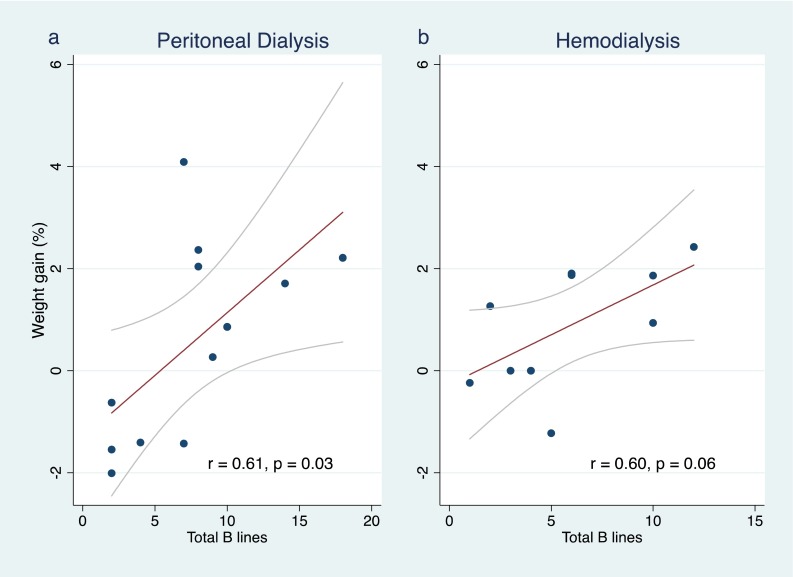
Correlation of B-lines on lung ultrasound with measured weight overload in patients receiving peritoneal dialysis and haemodialysis

The number of B-lines on lung ultrasound images was the single parameter with the strongest linear correlation with fluid overload by weight (*r* = 0.57, *p* = 0.005; Fig. 2). A similar positive correlation between B-lines and measured fluid overload was observed in both PD (*r* = 0.61, *p* = 0.03) and HD (*r* = 0.60, *p* = 0.06; Fig. 3). A non-significant but positive linear correlation with fluid overload by weight was also observed with the BIS measurement (*r* = 0.43, *p* = 0.2), systolic blood pressure (*r* = 0.19, *p* = 0.4) and clinical examination score (*r* = 0.19, *p* = 0.4; Fig. 2). There was a non-significant negative linear relationship with IVC collapsibility index (*r* = −0.24, *p* = 0.3; Fig. 2). No linear correlation was found with IVC diameter during inspiration and expiration or with mean IVC diameter (*r* = 0.08, −0.14 and −0.05, respectively; Table 2).

**Table 2:** Correlation of fluid parameters with proportional increase in weight from the prescribed target weight

Parameter	Correlation with fluid overload by weight	*p* value
B-lines on lung ultrasound images	0.57	0.005
Clinical examination score	0.19	0.4
Systolic blood pressure elevation above 50th centile	0.19	0.4
Bioimpedance spectroscopy	0.43	0.2
IVC diameter during inspiration	0.08	0.7
IVC diameter during expiration	−0.14	0.5
Mean IVC diameter	−0.05	0.8
IVC collapsibility index	−0.24	0.3

IVC, Inferior vena cava

**Fig. 4:**
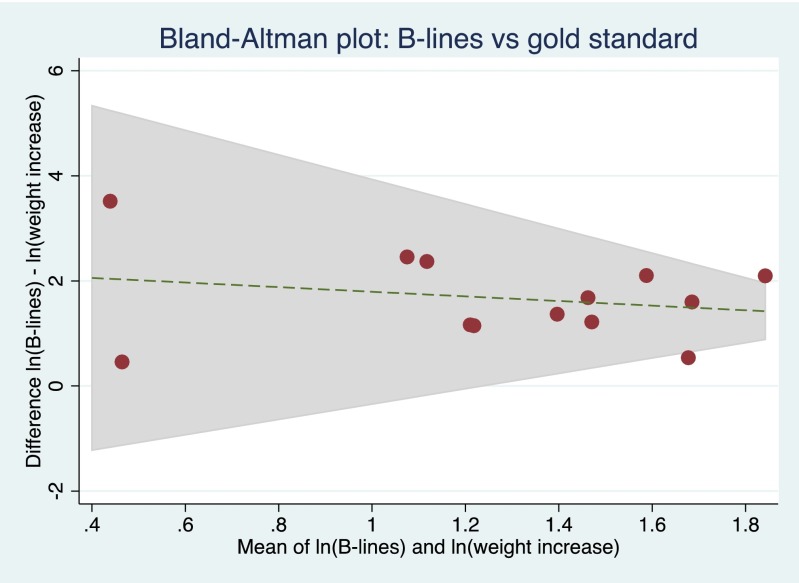
Bland–Altman analysis comparing B-lines on the lung ultrasound image to the gold standard of weight increase following natural logarithmic transformation to account for differences in scale between parameters with limits of agreement represented by the shaded area.

The sensitivity of the observed correlation between B-lines on the lung ultrasound images and measured weight overload to changes in the target weight was examined. No significant changes in the correlation were observed with up to 5 % reduction in the target weight (*r* = 0.57, *p* = 0.05 with reduction in target weight by 5 % of the measured weight). Bland–Altman analysis showed acceptable levels of agreement between B-lines and the gold standard comparator of fluid overload (Fig. 4).

A combination of three fluid parameters, namely B-lines, clinical examination and systolic blood pressure, best predicted fluid overload by weight in a multiple linear regression model (*R* ^*2*^ = 0.46, *p* = 0.05). The final model with beta coefficients is summarized below:

$$ Fluid\kern0.5em  overload\left( weight\kern0.5em \%\right)=0.55\ast Blines+0.07\ast clinicalexam+0.04\ast SBPincrement $$

where Blines =the total number of B-lines on the lung ultrasound image; clinicalexam = clinical fluid overload score (described above); SBPincrement = proportional increase in systolic BP above 50th centile (%).

**Fig. 5:**
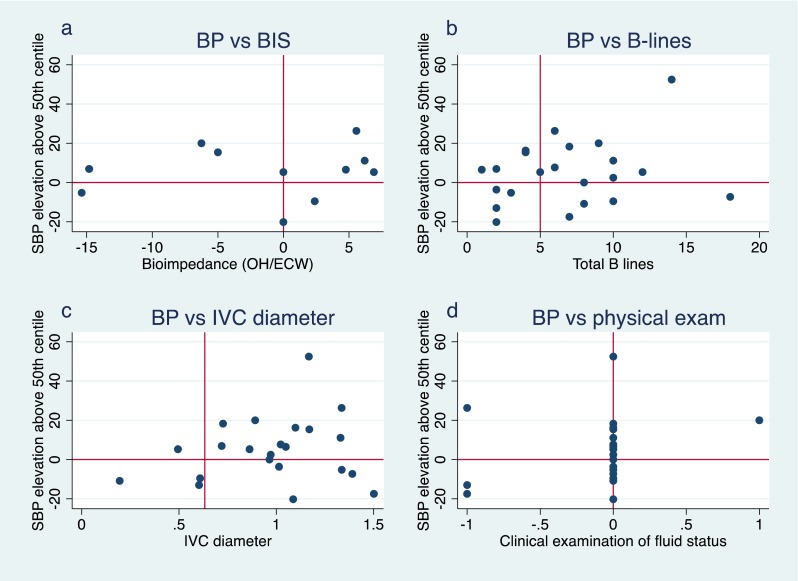
Dissociation between SBP elevation and measured parameters of fluid overload.* Vertical lines* Cutoff measurements for volume overload,* horizontal lines* 50th centile SBP. Dissociated readings are in the *upper-left* and* lower-right* quadrants of each plot, * SBP* Systolic blood pressure,* IVC* inferior vena cava, *BIS* Bioimpedance spectroscopy

The frequency of dissociation between BIS measurements and blood pressure was 36 %, with three of 11 measurements showing underhydration with elevated blood pressure, and one of 11 measurements showing overhydration with low blood pressure (Fig. 5a). For the lung ultrasound, four (18 %) of 22 readings showed more than five B-lines with low blood pressure (Fig. 5b). Six (27 %) IVC readings were discordant with blood pressure measurements (Fig. 5c). Physical examination showed the least dissociation with blood pressure in one (4 %) of 22 physical examination scores (Fig. 5d).

## Discussion

In this cohort of 13 infants and children with ESRD, fluid overload was best predicted using a combination of physical examination, blood pressure and lung ultrasound measurements. No significant predictive value from BIS or echocardiographic measurements was observed in the assessment of fluid overload in this study.

The BIS data from the present study contrast with previous reports of the utility of this technique in facilitating an accurate assessment of overhydration in children on dialysis [4–7, 23, 24]. In our study cohort, we observed a non-significant positive linear relationship between bioimpedance data and fluid overload as assessed by weight. The lack of a significant correlation between these two measurements may be related to limitations of the present study, such as small numbers compounded by a lower age limit for BIS of 2 years, as well as to limitations of the technique itself, such as clinically significant variation in measurements in children [25]. The impracticality of achieving ideal measurement conditions in children may also compromise the accuracy of this technique. Children on dialysis rarely lie completely still in a supine position; also, bladder volume and filling is variable in children. These and other factors specific to the paediatric patient population may explain the reduced reliability of bioimpedance in assessing fluid overload in children on dialysis as opposed to adults with ESRD [26].

Echocardiography is a well-established technique for fluid overload assessment in children. IVC diameter and collapsibility index were reported to be useful measures of overhydration in 14 children receiving HD as far back as two decades ago [27]. These findings were corroborated in a further study of 15 children [8]. Larger studies have subsequently allowed the development of reference nomograms for children with and without ESRD [9, 18]. Our data show a non-significant negative correlation between the IVC collapsibility index and fluid overload as assessed by weight, but no significant correlation between IVC diameter and weight overload. The lack of an observed relationship in our data may relate to limitations of this study, such as small numbers, a single-centre design and the lack of a robust gold standard measurement of fluid overload. In addition, IVC measurements can be influenced by systemic hypertension, pulmonary hypertension, diastolic dysfunction, myocardial infarction and valvular dysfunction. Moreover, congenital abnormalities of the IVC, such as interruption of the hepatic segment, azygous or hemiazygous continuation or duplication, can confound the IVC diameter measurement. Although these anomalies are uncommon, the technique described here may not be appropriate for all children. These limitations may underlie the discordance between the current data and the previously reported reliability of echocardiographic IVC measurements in studies with similar numbers.

The application of lung ultrasound imaging for the detection of generalized fluid overload in the dialysis population is receiving growing attention. In adult dialysis patients, the technique is reliable in quantifying subclinical fluid overload [11, 22, 28, 29], responsive to detect changes in real time immediately following dialysis ultrafiltration [30–32] and predictive of volume-related morbidity and mortality [33, 34]. We recently reported the value of lung ultrasound in detecting fluid overload in infants and children on dialysis [12]. In the current study, lung ultrasound findings were found to be the most useful objective predictor of fluid overload. We observed a significant linear correlation between lung ultrasound B-line measurements and fluid overload as assessed by weight. In addition, the technique was practical to undertake as a bedside test. Lung ultrasound examinations were straightforward and could be performed in less than 5 minutes using portable equipment. The technique was relatively easy to learn, requiring just 50 preparatory ultrasounds for both operators.

Physical examination is the mainstay of fluid assessment in the clinical setting; however, physical examination findings are subject to significant variability, even in the hands of experienced physicians [24]. Our data show some correlation between the physical examination score and fluid overload by weight, albeit less sensitive than other objective measures. While physical examination in isolation lacked power to quantify fluid overload, it did enhance the predictive value of objective measures when combined in a multiple linear regression model. The majority of children in this study were assessed as being clinically euvolaemic. In 61 % of all assessments in which children were clinically euvolaemic on physical examination, fluid overload was detected by objective measures. The poor sensitivity of the physical examination measurements could relate to limitations of this study in which a clinical fluid score with 5 integer points was used, which possibly lacked sufficient granularity to document more subtle degrees of volume overload. The superior sensitivity of objective techniques over physical examination in detecting fluid overload in children is more likely to be a genuine phenomenon and is well recognized in adult patients [22, 24, 28].

Hypertension is a well-established complication of ESRD in both adults and children [35]. While extracellular volume overload is often implicated in the aetiology of hypertension in children with ESRD, it is not the sole responsible factor [36–38]. The multifactorial aetiology of hypertension in ESRD may be the reason for the lack of a clear relationship between systolic blood pressure and volume overload observed in this study. Although systolic blood pressure considered in isolation lacked sensitivity to predict fluid overload, when taken in combination with the clinical examination and lung ultrasound findings, it enhanced the accuracy of prediction of fluid overload by weight.

The clinical significance of subclinical volume overload detected using techniques such as BIS remains open to debate in view of significant dissociation between hypertension and overhydration measurements in children [23]. Our data corroborate these findings in BIS, echocardiography and lung ultrasound. Notwithstanding these concerns, the clinical impact of sonographically detected volume overload in adult patients on dialysis is well established, namely cardiovascular morbidity and mortality [33, 34]. Although caution is required in extrapolating adult data to children, we suggest that subclinical volume overload detected by lung ultrasound imaging in children on dialysis may impact their cardiovascular health. Further work is needed to determine if optimization of target weight using lung ultrasound to complement clinical assessment improves cardiovascular outcomes, albeit with caution in younger children in whom hypotension is a particular risk when reducing target weight.

This study has a number of limitations. As a relatively small, single-centre study, it lacks the statistical power required to reach firm conclusions on the superiority of any fluid assessment technique. The study population is heterogeneous with respect to both patient age and dialysis modality. The gold standard measure of fluid overload, namely deuterium dioxide dilution, was not used due to ethical concerns about repeated blood sampling in infants and children. Notwithstanding these limitations, sufficient data relating to the practicality and utility of fluid assessment techniques are presented which allow their use in paediatric patients with ESRD to be compared for the first time.

The results of this study suggest that lung ultrasound imaging may be superior to both echocardiographic methods and BIS in detecting volume overload in children with ESRD. Our data demonstrate that lung ultrasound was the most practical of the three techniques, being undertaken with a portable device at the bedside in <5 minutes per examination. The technique is straightforward to learn for non-radiologists. It can be used in infants and babies under 2 years of age, for whom BIS is not validated. Given the practicality and sensitivity of lung ultrasound imaging, this technique can be adopted alongside clinical examination and blood pressure in the routine assessment of fluid overload in children with ESRD.
